# Intramuscular injection, intravenous infusion, and intravenous bolus of oxytocin in the third stage of labor for prevention of postpartum hemorrhage: a three-arm randomized control trial

**DOI:** 10.1186/s12884-019-2181-2

**Published:** 2019-01-18

**Authors:** Dyanna Charles, Holly Anger, Rasha Dabash, Emad Darwish, Mohamed Cherine Ramadan, Amr Mansy, Yomna Salem, Ilana G. Dzuba, Meagan E. Byrne, Miral Breebaart, Beverly Winikoff

**Affiliations:** 1 Gynuity Health Projects, 220 East 42nd St, Suite 710, New York, NY 10010 USA; 2 Shatby Maternity Hospital, Alexandria University, Alexandria, Egypt; 3 El Galaa Teaching Hospital, 41 El-Galaa, Cairo, Egypt; 4 Independent Consultant, 16B Dr. Mostafa el Diwani, Garden City, Cairo, Egypt

**Keywords:** Postpartum hemorrhage, Postpartum blood loss, Oxytocin, Oxytocic, Route of administration, Third stage of labor, Bolus oxytocin, Intravenous oxytocin, Intramuscular oxytocin, Oxytocin prophylaxis

## Abstract

**Background:**

Oxytocin for postpartum hemorrhage (PPH) prophylaxis is commonly administered by either intramuscular (IM) injection or intravenous (IV) infusion with both routes recommended equally and little discussion of potential differences between the two. This trial assesses the effectiveness and safety of 10 IU oxytocin administered as IM injection versus IV infusion and IV bolus during the third stage of labor for PPH prophylaxis.

**Methods:**

In two tertiary level Egyptian maternity hospitals, women delivering vaginally without exposure to pre-delivery uterotonics were randomized to one of three prophylactic oxytocin administration groups after delivery of the baby. Blood loss was measured 1 h after delivery, and side effects were recorded. Primary outcomes were mean postpartum blood loss and proportion of women with postpartum blood loss ≥500 ml in this open-label, three-arm, parallel, randomized controlled trial.

**Results:**

Four thousand nine hundred thirteen eligible, consenting women were randomized. Compared to IM injection, mean blood loss was 5.9% less in the IV infusion arm (95% CI: -8.5, − 3.3) and 11.1% less in the IV bolus arm (95% CI: -14.7, − 7.8). Risk of postpartum blood loss ≥500 ml in the IV infusion arm was significantly less compared to IM injection (0.8% vs. 1.5%, RR = 0.50, 95% CI: 0.27, 0.91). No side effects were reported in any arm.

**Conclusions:**

Intravenous oxytocin is more effective than intramuscular injection for the prevention of PPH in the third stage of labor. Oxytocin delivered by IV bolus presents no safety concerns after vaginal delivery and should be considered a safe option for PPH prophylaxis.

**Trial registration:**

clinicaltrials.gov #NCT01914419, posted August 2, 2013.

## Background

Active management of the third stage of labor (AMTSL) is recommended to prevent postpartum hemorrhage (PPH), with uterotonics considered the most important component [1], and oxytocin the uterotonic of choice [1–6]. In most hospital settings, oxytocin is used for this indication, but with much variation in route, dose, and timing [3, 7–10]. Oxytocin is commonly administered either intramuscularly (IM) or intravenously (IV). International guidelines, including the World Health Organization, currently recommend both routes equally [1, 11].

There are potential advantages to each route. IV administration may have a clinical advantage, as it leads to a faster response and a higher peak in plasma oxytocin levels [12–14]; however, IM injection confers practical advantages, requiring fewer skills and less equipment to administer, making it a more serviceable option in a wider array of settings [9, 15]. Despite many discussions of the clinical importance of route [9, 15–25], differences in efficacy remain largely uninvestigated. The few published studies investigating route are inconsistent, with two showing reduced blood loss associated with IV administration [24, 25] and two others showing no difference between IV and IM administration [20, 21].

Clinical effects may also be different if intravenous oxytocin is delivered via bolus push or over a longer duration via dilute infusion. While there is some evidence that the more immediate, higher concentration of bolus delivery could lead to stronger effect on uterine contractions [23], this route is less frequently used due to fear of hypotension, although this problem has only been noted in case studies of women under general anesthesia during caesarean section [26–28]. Two studies conducted among women who received oxytocin following vaginal delivery showed no side effects or adverse outcomes associated with IV bolus administration and somewhat worse clinical outcomes associated with IV infusion [22, 23]. Despite this evidence, hesitation persists regarding oxytocin via IV bolus.

To help inform best practices in clinical care and address inconsistencies and gaps in the literature, we conducted a three-arm study to compare the clinical effectiveness and safety of IM injection to both IV infusion and IV bolus of 10 IU oxytocin administered during the third stage of labor.

## Methods

Pregnant women presenting for vaginal delivery in two tertiary Egyptian hospitals were screened for participation in this open-label, three-arm, parallel, randomized controlled trial. Approval was obtained from the research ethics committees of both hospitals: El Galaa Teaching Hospital in Cairo (the largest maternity hospital in Cairo), and Shatby Maternity Hospital in Alexandria (the university hospital of Alexandria University), where all three routes of oxytocin administration were routinely used.

Women were eligible to participate if they delivered a live birth vaginally, did not receive pre-delivery uterotonics to induce or augment labor, and were able to provide informed consent. Written consent was obtained after admission, upon arrival to the labor ward. Blood pressure and pre-delivery hemoglobin were subsequently measured and recorded, the latter using HemoCue® hb 201+ (HemoCue, Ängelholm, Sweden).

Women were randomized to receive 10 IU of oxytocin by IM injection, IV infusion, or IV bolus immediately after delivery of the baby. IM injection was usually administered in the thigh. For IV infusion, oxytocin was mixed in 500 ml of fluid and administered through gravity-driven infusion with the roller clamp fully open, most often using an 18 gauge needle. IV bolus was pushed directly into the IV port over approximately 1 min.

Information on other prophylactic measures provided in the third stage of labor, including controlled cord traction and uterine massage, was recorded on standardized data collection forms. Postpartum blood pressure and any side effects or adverse events experienced after oxytocin administration were also recorded. Postpartum blood loss was measured at 1 h post-delivery using a plastic blood collection drape funneled into a calibrated container. For women diagnosed with PPH, blood loss was also recorded at the time of PPH diagnosis and at active bleeding cessation. Women diagnosed with PPH received standard of care treatment at each hospital. Interventions, including administration of additional uterotonics or blood transfusion, were documented. Postpartum hemoglobin was measured at least 24 h after delivery and at least 12 h after removal of the IV for women receiving IV fluids, if possible, or just before discharge if women were discharged sooner.

Our primary outcomes were mean blood loss and proportion of women with blood loss ≥500 ml. Secondary outcomes included proportion of women with blood loss ≥350 ml and ≥ 1000 ml, change in pre- to post-delivery hemoglobin, time to placental delivery, administration of additional oxytocin or other uterotonics, and observed side effects within 1 h postpartum.

The sample size calculation was derived from the expected rate of women with blood loss ≥500 ml in the two comparisons of this three-arm study: IM injection vs. IV infusion and IM injection vs. IV bolus. Based on previous studies, we expected a slightly larger difference of blood loss outcomes for the IV bolus vs. IM injection comparison, thus a smaller sample size was required for that comparison [23, 29]. We augmented sample sizes to compensate for conducting two 80% correlated comparisons (equivalent to requiring a significance level of 0.0435 for each test) and to account for a 2% attrition rate. The resulting sample size requirement was 4900 women, at a 3:3:1 ratio (2100 in each of the IM injection and IV infusion groups and 700 in the IV bolus group), with 80% power for the comparison of IM injection to IV infusion and 85% power for the comparison of IM injection to IV bolus administration. The sample size was also sufficient to detect a 50 ml mean difference in blood loss between study groups.

The simple randomization code was computer-generated in blocks of seven at Gynuity Health Projects in New York, and each assignment was contained in a sequentially numbered, sealed, opaque envelope. Each hospital was independently randomized. Hospital study staff had no access to the randomization code and were instructed to open the next envelope prior to the woman’s delivery, during the second stage of labor.

Analysis was done using the intent to treat approach. *P* values for baseline characteristics were calculated using the chi-square test of association for categorical variables and one-way analysis of variance (ANOVA) for continuous variables. Differences were considered significant at α = 0.0435, to account for the multiple comparisons made in this three-arm study. Log-binomial regression was used to calculate relative risks (RRs) and associated 95% confidence intervals (CIs) for categorical outcomes. Linear regression was used to calculate regression coefficients and associated 95% CIs for continuous outcomes. We first assessed the assumption of normal distribution all continuous secondary outcomes (including postpartum blood loss, time to placental delivery in minutes, total blood loss, and change in pre-to post-delivery hemoglobin). None were normally distributed, thus transformation (using the natural log ln) was done on all continuous outcomes. To facilitate interpretation of estimates obtained from linear regression of these log-transformed outcomes, we used the following formula to produce an estimate of the percent change in the mean outcome (y) associate with treatment group in question (d): y = 100·[exp(βd) − 1], where β is equal to the regression coefficient for the log-transformed outcome. Analyses were conducted using Stata 12 (StataCorp. 2011. *Stata Statistical Software: Release 12.* College Station, TX: StataCorp LP).

## Results

**Fig. 1:**
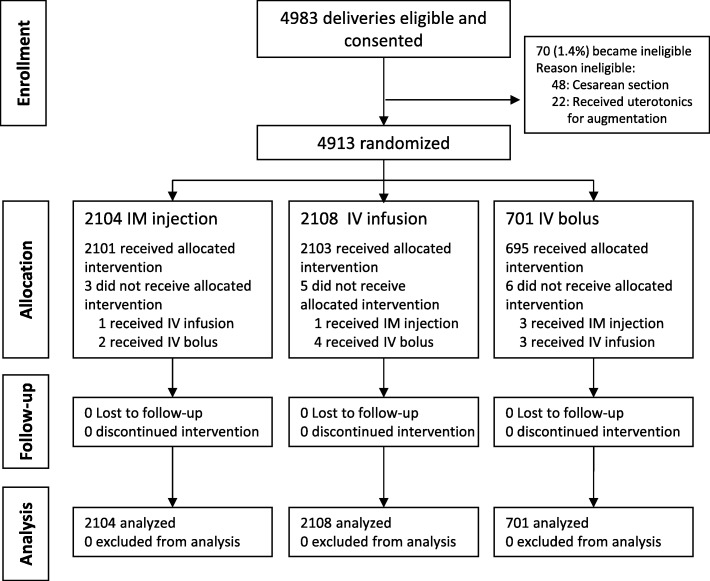
CONSORT diagram

Between April 2014 and September 2015, 4983 women with eligible deliveries were screened and enrolled in the study from 15,143 total vaginal deliveries at El Galaa Teaching Hospital and 8353 total vaginal deliveries at Shatby Maternity Hospital. Recruitment ended when the target sample size was confirmed achieved. Of those enrolled, 70 (1.4%) were not randomized due to ineligibility before delivery (Fig. 1). All women randomized were included in the analysis. Of the 4913 women randomized, 2104 were randomized to receive prophylactic oxytocin via IM injection, 2108 via IV infusion, and 701 via IV bolus. In each group, there were few cases (< 1% in all groups) who had oxytocin administered via a route different from the assigned route.

Women randomized to each of the three arms were similar with respect to demographic and delivery characteristics except for episiotomy which, although done before oxytocin administration, was less common among women randomized to IM injection administration (Table 1). The mean time to completion of the 500 ml infusion for women randomized to the IV infusion arm was 28 min (SD = 6.4).

**Table 1:** Demographic and delivery characteristics among women randomized to one of three routes of oxytocin administration

	IM injection (*n* = 2104)	IV infusion (*n* = 2108)	IV bolus (*n* = 701)	*p*-value*
DEMOGRAPHICS				
Age, mean (SD)	27 (5.3)	27 (5.3)	26 (5.2)	0.074
Education, % (n)				
None	27.7 (582)	28.9 (609)	27.4 (192)	0.565
Primary	12.4 (261)	10.8 (227)	13.7 (96)	
Preparatory	18.8 (395)	20.5 (433)	18.8 (132)	
Secondary	6.1 (129)	6.1 (128)	6.8 (48)	
Technical	27.6 (581)	26.7 (563)	26.0 (182)	
University	7.4 (156)	7.0 (148)	7.3 (51)	
Marital status, % (n)				0.176
Married	99.5 (2093)	99.8 (2103)	99.9 (700)	
Widowed/divorced	0.5 (11)	0.2 (5)	0.1 (1)	
DELIVERY CHARACTERISTICS				
Mean Hb at enrollment (SD)	11.4 (1.15)	11.4 (1.17)	11.4 (1.11)	0.481
Gestational < 37 weeks, % (n)	10.7 (226)	11.8 (248)	11.6 (81)	0.561
> 3 previous live births, % (n)	17.1 (360)	15.4 (324)	15.3 (107)	0.249
Nulliparous, % (n)	32.4 (682)	32.0 (674)	30.0 (210)	0.477
Known previous PPH, % (n)	0.5 (11)	0.8 (17)	0.7 (5)	0.523
Multiple birth, % (n)	1.4 (29)	1.2 (25)	2.1 (15)	0.176
Epidural, % (n)	0.6 (12)	1.2 (26)	0.7 (5)	0.061
Episiotomy, % (n)	**39.3 (826)**	**44.1 (930)**	**44.5 (312)**	**0.002**
Controlled cord traction, % (n)	93.9 (1975)	94.5 (1992)	95.3 (668)	0.339
Uterine massage, % (n)	89.4 (1880)	89.0 (1876)	89.3 (626)	0.927

*p values derived from chi-square test for categorical variables and one-way ANOVA test for continuous variables

In bold: comparison statistically significant at *p* ≤ 0.0435

### Primary outcomes

Postpartum blood loss was significantly lower after both IV infusion and IV bolus than after IM injection. Compared to women randomized to oxytocin administration via IM injection, mean postpartum blood loss was 5.9% less in those randomized to IV infusion (95% CI: -8.5, − 3.3) and was 11.1% less in those randomized to IV bolus administration (95% CI: -14.7, − 7.8, Table 2). The risk of having postpartum blood loss ≥500 ml among women receiving oxytocin via IV infusion was significantly reduced compared to women receiving IM injection oxytocin (0.8% vs. 1.5%, RR = 0.50, 95% CI: 0.27, 0.91). The risk was also lower with IV bolus compared to IM injection (1.0% vs. 1.5%, RR = 0.66), though not statistically significant (95% CI: 0.29, 1.48).

**Table 2:** Primary and secondary outcomes among 4913 women randomized to one of three routes of oxytocin administration during the third stage of labor

		Comparison of IV infusion to IM injection			Comparison of IV bolus to IM injection		
	IM injection (*n* = 2104)	IV infusion (*n* = 2108)	Estimate^a^ (95% CI)	P value	IV bolus (n = 701)	Estimate^a^ (95% CI)	P value
Primary outcomes							
Mean total blood loss (SD)	**204 (117)**	**188 (90)**	**−5.9 (−8.5, −3.3)** ^**b**^	**< 0.001**	**180 (104)**	**−11.1 (−14.7, −7.8)** ^**b**^	**< 0.001**
Blood loss ≥500 ml, % (n)	**1.5 (32)**	**0.8 (16)**	**0.50 (0.27, 0.91)**	**0.023**	1.0 (7)	0.66 (0.29, 1.48)	0.311
Secondary outcomes							
Mean min to placenta delivery (SD)	**6.0 (3.8)**	**6.2 (4.1)**	**5.2 (1.5, 9.0)** ^**b**^	**0.006**	5.6 (3.4)	−4.9 (−9.7, − 0.1)^b^	0.047
Postpartum blood loss, % (n)	N = 2104	N = 2108			N = 701		
≥ 350 ml	**7.7 (163)**	**4.4 (92)**	**0.56 (0.44, 0.72)**	**< 0.001**	**4.0 (28)**	**0.52 (0.35, 0.76)**	**0.001**
≥ 1000 ml	0.4 (9)	0.2 (4)	0.44 (0.14, 1.44)	0.176	0.1 (1)	0.33 (0.04, 2.63)	0.297
PPH diagnosed, % (n)	1.1 (22)	0.6 (12)	0.54 (0.27, 1.10)	0.089	0.9 (6)	0.82 (0.33, 2.01)	0.662
PPH diagnosed + ≥500 ml blood loss, % (n)	1.0 (21)	0.5 (11)	0.52 (0.25, 1.08)	0.080	0.7 (5)	0.71 (0.27, 1.89)	0.498
Hemoglobin measures^c^	*N* = 2094	*N* = 2103			*N* = 700		
Change in Hb, mean (SD)	−0.54 (0.50)	− 0.54 (0.46)	0.02 (− 0.29, 0.35)^b^	0.876	−0.51 (0.45)	0.29 (− 0.16, 0.75)^b^	0.209
Drop in Hb ≥2 g/dl, % (n)	2.1 (44)	1.8 (38)	0.86 (0.56, 1.32)	0.491	1.4 (10)	0.68 (0.34, 1.34)	0.267
Additional interventions, % (n)	N = 2104	N = 2108			N = 701		
Manual removal of placenta	**2.9 (60)**	2.4 (50)	0.83 (0.57, 1.20)	0.330	**1.3 (9)**	**0.45 (0.22, 0.90)**	**0.024**
Additional uterotonics	1.1 (23)	0.6 (13)	0.56 (0.29, 1.11)	0.098	1.0 (7)	0.91 (0.39, 2.11)	0.833
Blood transfusion	0.5 (10)	0.2 (5)	0.50 (0.17, 1.46)	0.204	0.1 (1)	0.30 (0.04, 2.34)	0.251

^a^Estimate reflects relative risk (RR) generated from log-binomial regression, except where otherwise noted

^b^Estimates reflect the percent change in the mean outcome over different treatment groups – these were generated using linear regression on the log-transformed outcome and then applying the eq. )*β*[exp(·100 − *β*1], where *=* the regression coefficient for the log-transformed outcome

^c^Excludes women who received blood transfusion

In bold: comparison statistically significant at *p* ≤ 0.0435

### Secondary outcomes

Compared to women randomized to IM injection, women in the IV infusion group (RR = 0.56, 95% CI: 0.44, 0.72, Table 2) and the IV bolus group (RR = 0.52, 95% CI: 0.35, 0.76) were significantly less likely to have blood loss ≥350 ml. In addition, manual removal of the placenta was significantly less likely among women randomized to IV bolus administration compared to women in the IM injection group (RR = 0.45, 95% CI: 0.22, 0.90). Among the other secondary outcomes, there were lower occurrences of blood loss ≥1000 ml, PPH diagnosis, pre- to post-delivery hemoglobin drop ≥2 g/dL, and use of additional uterotonics for PPH management after both IV infusion and IV bolus administration as compared to IM injection, though these outcomes were rare and differences were not statistically significant (Table 2).

### Adverse effects

No notable side effects or adverse effects were reported in any of the three intervention arms, including no reports of intensive care admission, shock, or death. Blood pressure measurements 1 h after delivery were similar in the IM injection (mean systolic = 113.7, mean diastolic = 73.1), IV infusion (mean systolic = 113.7, mean diastolic = 73.4) and IV bolus (mean systolic = 113.1, mean diastolic = 72.9) groups with no statistically significant differences (systolic *p* = 0.236, diastolic *p* = 0.192). Similarly, no statistically significant differences were seen in proportion of hypotension (systolic pressure ≤ 90, diastolic pressure ≤ 60 mmHg) across groups 1 h after delivery (IM injection (systolic = 0.4%, diastolic = 10.0%), IV infusion (systolic = 0.6%, diastolic = 10.0%) and IV bolus (systolic = 0.4%, diastolic = 9.0%)).

## Discussion

The findings of this large randomized controlled trial exploring difference in route of prophylactic oxytocin administration in the third stage of labor suggest that route of oxytocin administration affects postpartum blood loss. These results substantiate earlier findings [12, 13, 24] that both IV infusion and IV bolus administration of 10 IU of oxytocin were associated with significantly less average postpartum blood loss when compared to IM injection.

This trial is one of few studies to include the less commonly studied IV bolus administration after vaginal delivery. Our trial found no safety issues with any of the oxytocin administration routes, including IV bolus. Obstetrical practice moved away from IV bolus based upon reports of hemodynamic effects exhibited after oxytocin administration by IV bolus in women undergoing general anesthesia for cesarean section; however, our study corroborates more recent reports that these concerns are unnecessary for vaginal deliveries [22, 23].

Because the total difference in average blood loss (24 ml) was small, the clinical application of these results may be limited; however, our findings have important implications for research on PPH prevention. For example, based on existing data, current World Health Organization guidelines recommend IV and IM administration equally for the prevention of PPH and strongly recommend use of the non-parenteral option, misoprostol, only in settings where use of oxytocin is not possible [1]. However, the key studies underlying international guidelines [30, 31] are based upon data with all routes of oxytocin administration combined. As our study clearly demonstrates the need to disaggregate such findings by route of administration, there is no clear evidence that IM injection of oxytocin is superior to other uterotonics for PPH prevention. If data from key studies that underpin WHO recommendations were disaggregated by oxytocin route, a more robust comparison of IM injection with misoprostol would be possible. As IM oxytocin and misoprostol are the most practical options for PPH prophylaxis in low resource settings, the longer shelf life and greater stability of misoprostol [32–35] could make it a preferable option if the two modalities were found to be equivalent [32].

The large size of this trial ensured that we could detect differences between IV and IM routes. Exclusion of women who had received uterotonics for induction/augmentation of labor made it easier to clearly assess the impact of route of administration on blood loss during the third stage of labor, since pre-delivery oxytocin can desensitize the uterus to the effect of subsequent doses [36], though results may be less generalizable to these women.

### Limitations

This study is not blinded because blinding would create additional burden for both women and providers by requiring unnecessary IV lines and injections to be administered. We minimized provider bias by having staff other than the administering provider assess blood loss using calibrated containers for objective measurement. Additionally, electric pumps were not available at these sites. While this made it more difficult to strictly define IV infusion rate, we prioritized reporting results reflective of the standard of care currently in practice at these hospitals and in other comparable settings around the world. To help standardize infusion rates, sites did receive uniform instructions for the IV set-up and gauge of the needle.

## Conclusion

Route of oxytocin administration should be standardized and specified in research design and interpretation, and different routes cannot be presumed to be equivalent. Recommendations on drugs for prevention of PPH should consider route of administration when ranking prophylaxis options for future guidelines.

For clinical practice, providers might benefit from knowing 10 IU of IV bolus is a good, safe option for women after vaginal delivery. If an IV line is already in place at delivery, IV infusion or IV bolus administration of oxytocin may be preferable to IM injection in the third stage of labor.

## Abbreviations

AMTSL: Active management of the third stage of labor
IM: Intramuscular
IV: Intravenous
PPH: Postpartum hemorrhage
